# An Indoor Localization System Using Residual Learning with Channel State Information

**DOI:** 10.3390/e23050574

**Published:** 2021-05-07

**Authors:** Chendong Xu, Weigang Wang, Yunwei Zhang, Jie Qin, Shujuan Yu, Yun Zhang

**Affiliations:** 1 College of Electronic and Optical Engineering, Nanjing University of Posts and Telecommunications, Nanjing 210023, China; 1219023433@njupt.edu.cn (C.X.); 1218022904@njupt.edu.cn (Y.Z.); 1218022905@njupt.edu.cn (J.Q.); yusj@njupt.edu.cn (S.Y.); y021001@njupt.edu.cn (Y.Z.); 2 National and Local Joint Engineering Laboratory of RF Integration and Micro-Assembly Technology, Nanjing 210023, China

**Keywords:** indoor localization, channel state information (CSI), denoising neural network (NN), residual network (ResNet)

## Abstract

With the increasing demand of location-based services, neural network (NN)-based intelligent indoor localization has attracted great interest due to its high localization accuracy. However, deep NNs are usually affected by degradation and gradient vanishing. To fill this gap, we propose a novel indoor localization system, including denoising NN and residual network (ResNet), to predict the location of moving object by the channel state information (CSI). In the ResNet, to prevent overfitting, we replace all the residual blocks by the stochastic residual blocks. Specially, we explore the long-range stochastic shortcut connection (LRSSC) to solve the degradation problem and gradient vanishing. To obtain a large receptive field without losing information, we leverage the dilated convolution at the rear of the ResNet. Experimental results are presented to confirm that our system outperforms state-of-the-art methods in a representative indoor environment.

## 1. Introduction

Due to the large demand for indoor localization, it attracts plenty of attention as an emerging technology. In the past, some indoor localization schemes based on WiFi, Bluetooth, RFID et al. have been proposed. Among them, indoor localization based on WiFi promises to become a large scale implemented technology. This is because the widespread deployment of WiFi access points (APs) enables users to obtain their locations at anytime and anywhere in public places. Various WiFi-based indoor localization schemes mainly fall into four categories: angle of arrival-based [1], time of arrival-based [2], signal propagation model-based [3], and fingerprint-based [4]. Since the fingerprint-based localization has a superior performance, it becomes the hot-pot of research.

Because received signal strength (RSS) is relatively easy to be measured and used [5], it has been utilized as fingerprint in many existing methods. The first fingerprint system based on RSS, named Radar, utilized a deterministic method for location estimation [6]. Horus utilized a probabilistic method for indoor localization with RSS values [7], which achieves better localization accuracy than Radar. However, for the impact of multipath effects, RSS fluctuates greatly over time in the same location. In addition, RSS does not exploit the rich channel information from different subcarriers. Thus, RSS-based fingerprint system is hard to satisfy the requirements for high localization accuracy.

Recently, an alternative fingerprint, termed CSI in the IEEE 802.11 standard [8], is applied to indoor localization. We can obtain CSI from some advanced WiFi network interface cards (NICs) and extract fine-grained information from its amplitude and phase. Compared to RSS, CSI has better time stability and location discrimination. With the great achievement of Deep Learning, many indoor fingerprint systems, based on neural networks, have been proposed for localization. DeepFi [9,10] learned 90 CSI amplitude data from three antennas for indoor localization and trained the deep network with a greed learning algorithm. However, there were too many network parameters to be trained and stored, which limits its application. Different from DeepFi, ConFi [11] converted the CSI data into CSI images and formulated indoor localization as a classification problem. The CSI images were fed into a five-layers convolutional neural network (CNN) to obtain features. Convolution operation in ConFi effectively improved the localization accuracy in the indoor scenario. However, the degradation problem and gradient vanishing will be caused due to the increasing depth of CNN.

Compared to CNN, ResNet has a superior performance in image classification [12], object detection [13], instance segmentation [14], etc. As we know, ResNet1D [15] utilized ResNet for indoor localization and outperformed ConFi in localization accuracy. Unfortunately, ResNet1D has a poor ability of feature expression and network convergence. Thus, in this paper, we propose a ResNet-based indoor WiFi localization scheme that uses CSI amplitude as feature. In our scheme, the raw CSI amplitude information is first extracted from three wireless links. Then, we convert the amplitude information into CSI amplitude images and use them to train a 50 layers ResNet which has a good ability of feature propagation and can solve degradation problem well.

Although Zhou et al. [16] proved that noise enables the algorithm converge to a global optimum, we still have the necessity to perform denoising at first. According to [17,18], due to sensitivity of the raw amplitudes of CSI to noise, localization is severely disturbed by the ubiquitously random noise. The goal of image denoising is to obtain a clean image from a noisy image. Most existing methods, based on deep learning, utilize many pairs of noisy/clean images as training samples. However, it is difficult to obtain clean CSI images. This is because the noise, especially Gaussian, always exists in wireless links. Recently, some research is conducted to train the denoising NN with only noisy images. The Noise2Noise (N2N) method [19] used many pairs of noisy images with the same scene to train a denoising NN model. However, it is still difficult to collect extensive image pairs. Self2Self (S2S) [20] was proposed to remove the noise by using Bernoulli sampled instances of an input noisy image. Although S2S greatly reduces the difficulty of collecting image pairs, it does not make full use of the low-level features, such as edge, colour, and pixels. Based on above, it is very urgent to explore a new method to cover these shortcomings.

The contributions of this paper can be summarized as follows:

(1) We design a novel residual network and solve the degradation problem effectively. All the ordinary residual blocks are replaced by the proposed stochastic residual blocks which can prevent overfitting.
(2) Meanwhile, we add long-range stochastic shortcut connections (LRSSCs) to alleviate gradient vanishing and strengthen feature propagation.
(3) Since some information may be lost in convolution and pooling layers, we use dilated convolution on the small size layer to gain a larger receptive field with low cost of memory.
(4) We elaborate a denoising NN to make it suitable for learning clean images. By leveraging the concatenation operation, we can further improve the denoising performance. Meanwhile, since the deep layers reuse the features learned from the shallow layers, we can reduce the parameters of deep layers.

## 2. Related Works

### 2.1. Channel State Information

The main idea of orthogonal frequency division multiplexing (OFDM) is to divide the channel into several orthogonal subchannels, which can reduce the mutual interference between the subchannels. By using the Intel 5300 NIC [21] or the Atheros AR9390 chipset [22], we can obtain CSI from the subchannels which reveals the channel characteristics. For OFDM system, the WiFi channel at the 2.4 GHz band can be regarded as a narrowband flat fading channel. The channel model is defined as

(1) R=HT+G,

where R and T represent the received and transmitted signal, respectively. G is the additive white Gaussian noise. H represents the channel frequency response(CFR). Ignoring the G, it can be calculated by

(2) $$H=\frac{R}{T}.$$

The CFR of the ith subcarrier can be represented as

(3) $$H_{i}=\left|H_{i}\right|e^{j∠H_{i}},$$

where $\left|H_{i}\right|$ and $∠H_{i}$ are the amplitude and phase response of the ith subcarrier, respectively. Generally, since the random noise and unsynchronized time clock between transmitter and receiver make the phase measurement has a large error, we only use amplitude as the fingerprint in this paper.

### 2.2. Image Denoising

We can only collect one noisy image at the same time and place, but traditional non-learning based methods cannot handle the denoising problem with it. Recently, some learning-based methods are proposed to solve this problem. Deep image prior (DIP) [23] showed that the structure of a convolutional image generator can get a large number of image statistics instead of learning. Although the algorithm and network model are simple, the optimal iteration number is hard to determine and the performance is unsatisfactory. S2S was proposed for image denoising using Bernoulli sampled instances which include the major information of the noisy image. By using Bernoulli dropout for reducing the variance of the prediction, the output of S2S gradually approximates to the clean image. Furthermore, in order to overcome the shortcoming of S2S in using low-level features insufficiently, we combine the low-level feature maps with multiple deep layers. By reusing the low-level features, we can obtain abundant background information.

### 2.3. ResNet

ResNet was firstly introduced in [12] to address the degradation problem. The bottleneck architecture, using a stack of three convolutional layers and one shortcut connection, was designed to fit a residual mapping. The first 1×1 layer is adopted to reduce dimensions, so that the 3×3 layer will have smaller input/output dimensions. Massive experiments show that this architecture can reduce the time complexity and model size. To obtain one-dimensional CSI fingerprint, ResNet was converted into ResNet1D. In order to retain the features of raw CSI and improve the model performance, the network uses pooling layer only in the input and output layer.

The degradation problem could also be largely addressed by batch normalization (BN) [24], which ensures forward propagated signals with non-zero variances. The success of ResNet is attributed to the hypothesis that residual mapping is easier to fit than original mapping. Furthermore, suppose that nested residual mapping is easier to fit than original residual mapping. Hence, we add several shortcut connections to alleviate the degradation problem and strengthen information propagation.

## 3. Localization System

**Figure 1:**
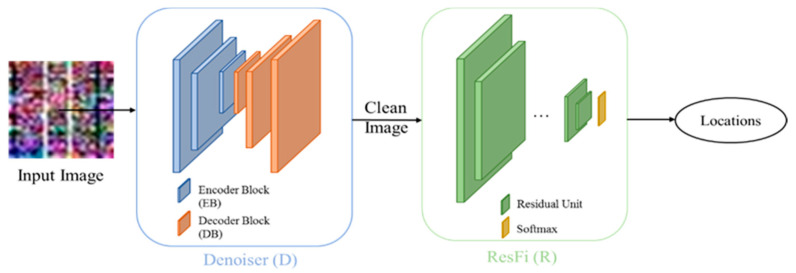
Pipeline of our system.

The two main networks of our system are illustrated in Figure 1. The “Denoiser” network works as a denoising NN which outputs a clean image, and the “ResFi” network works as a classification NN which outputs the corresponding location of a CSI amplitude image.

The input is a noisy CSI image, and we can get a clean image by removing the noise from it. After denoising, we can classify clean images by the ResFi. The design of Denoiser as well as ResFi will be elaborated in Section 3.

### 3.1. CSI Image Construction

**Figure 2:**
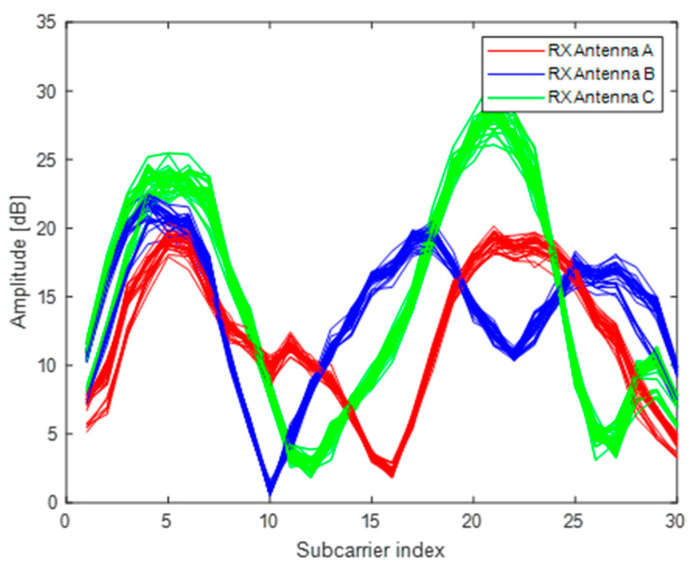
CSI amplitude of three different antennas in the same location.

**Figure 3:**
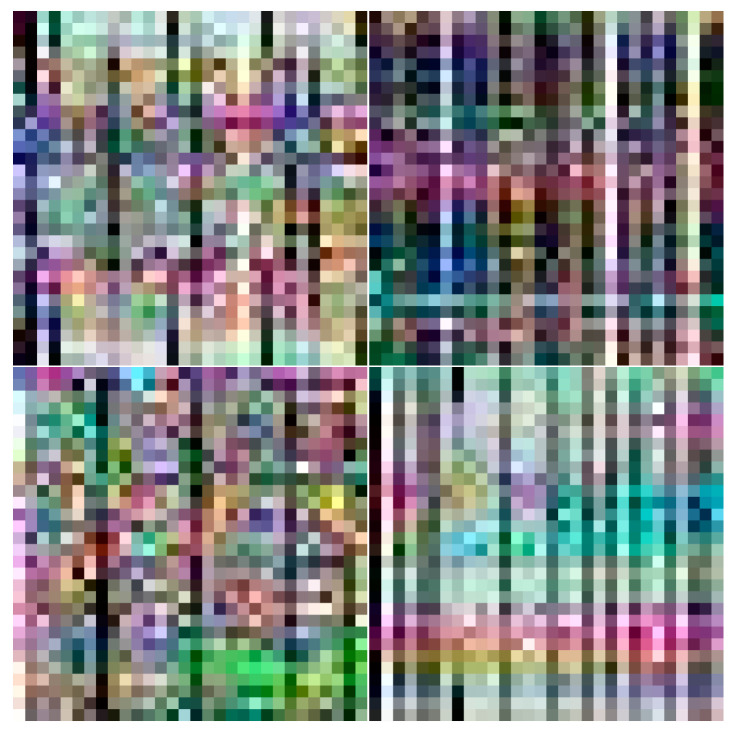
CSI images in different locations.

An Intel WiFi link (IWL) 5300 NIC which can read the CSI values of 30 subcarriers from 56 subcarriers is used as the received equipment, and a TP-Link wireless router is used as the transmitted equipment. Since only one antenna of wireless router is utilized, there are three wireless links between transmitter and receiver. We obtain 90 CSI data of three wireless links in a packet collection. For one wireless link, we take N packets in the same location and convert the CSI data as one channel of a RGB image. Thus, we can construct the RGB image by utilizing the CSI data of three wireless links. We set the N to 30000, and conveted the packets into 1000 images. As shown in Figure 2, the curves of three colors represent CSI data from three wireless links and the curve of each color is composed of 30 packets. The horizontal axis denotes the 30 subcarriers of a wireless link, and the vertical axis denotes the amplitude of CSI value. Figure 3 illustrates the CSI images in four different locations. The different data distributions of CSI images indicate that CSI images can be used as fingerprints for localization.

### 3.2. Modification of S2S

**Figure 4:**
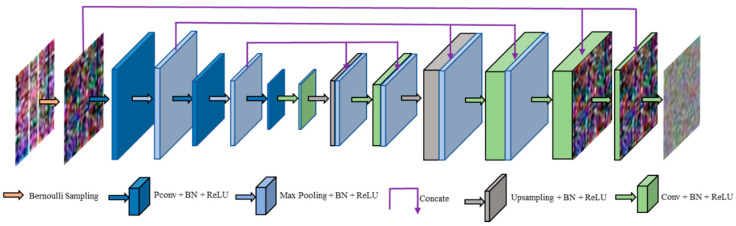
The architecture of modified S2S.

The architecture of modified S2S is shown in Figure 4. Given a noisy CSI amplitude image with the size of 30×30×3, we firstly utilize Bernoulli sampling to obtain a set of image pairs ${\left\{{\hat{n}}_{m},{\bar{n}}_{m}\right\}}_{m=1}^{M}$, and then, ${\hat{n}}_{m}$ is processed by the following three encoder blocks (EBs). The first two EBs are composed of a partial convolutional (PConv) layer [25] and a max pooling layer, respectively. The last EB is composed of only a PConv layer. We use the rectified linear unit (ReLU) [26] as the activation function. The number of channels of all EBs increases from 32 to 64, and then to 128. The output of the last EB is a feature map with size of 8×8×128.

After the EBs, there are three decoder blocks (DBs). The first DB is composed of a convolutional (Conv) layer, an upsampling layer, a Conv layer and a concatenation (Concate) operation. The second DB is composed of an upsampling layer, a Conv layer and a Concate operation. The last DB is composed of three Conv layers to map the layer to an image of size 30×30×3.The number of output channels of these Conv layers are 48, 24 and 3, respectively. For the low-level tasks, such as denoising, it is necessary to make full use of low-level features. Inspired by DenseNet [27], the Concate operation combine a low-level feature map with two deep layers. We use low-level features in deep layers two times and improve the information flow between layers by adding connections. Moreover, because we can reduce learning redundant feature maps in deep layers by feature reuse, this network requires fewer parameters than S2S.

**Figure 5:**
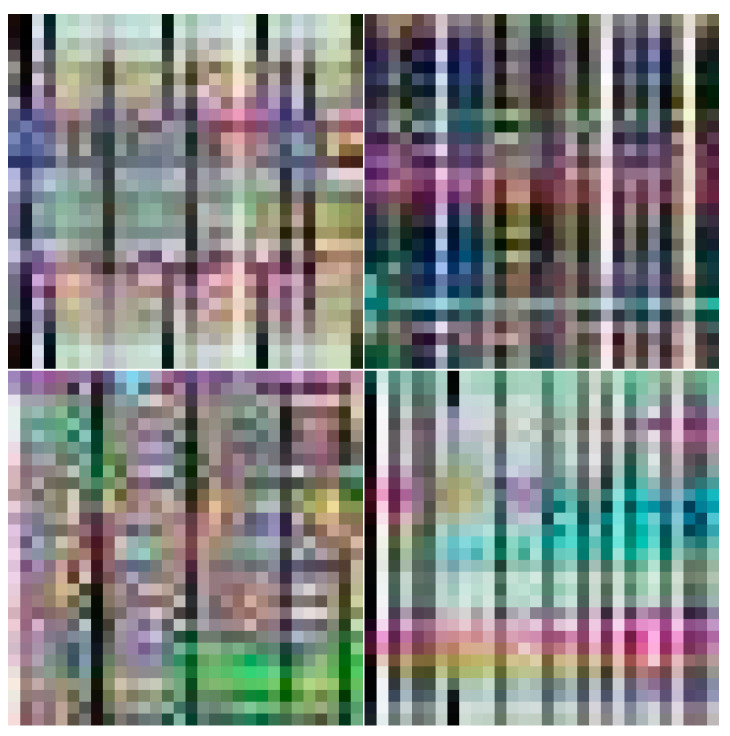
The denoised results.

Similar to S2S, we first sample a set of image pairs ${\left\{{\hat{n}}_{m},{\bar{n}}_{m}\right\}}_{m=1}^{M}$ from n, and they are defined as

(4) $${\hat{n}}_{m}:=b_{m}⊙n;\;\;{\bar{n}}_{m}:=\left(1-b_{m}\right)⊙n.$$

then the training objective $L_{D}\left(\theta \right)$ can be formulated by the mean squared error

(5) $$L_{D}(\theta )=\sum_{m=1}^{M}{\left\|b_{m}⊙{ℱ}_{\theta }\left({\bar{n}}_{m}\right)-{\hat{n}}_{m}\right\|}_{2}^{2},$$

where ⊙ denotes the elementwise multiplication. The loss of each image pair is calculated only on those pixels that are not eliminated by $b_{m}$. Since we use the Bernoulli sampling to randomly select pixels, the sum of loss of all pairs calculates the difference over all image pixels, and the expectation of $L_{D}\left(\theta \right)$ about noise is the same as

(6) $$\sum_{m=1}^{M}{\left\|{ℱ}_{\theta }\left({\bar{n}}_{m}\right)-x\right\|}_{b_{m}}^{2}+\sum_{m=1}^{M}{\left\|\delta \right\|}_{b_{m}}^{2},$$

where ${\left\|\cdot \right\|}_{b_{m}}^{2}={\left\|b_{m}⊙\cdot \right\|}_{2}^{2}$ and δ denotes the standard deviation of noise. When enough image pairs are used for training, the Denoiser will learn a clean image from the noisy image n. The denoised results corresponding to Figure 3 are displayed in Figure 5. We can observe that only the main line features have been preserved and the random noise has been well removed.

### 3.3. Structure of the ResFi

CNN has an outstanding performance in image classification [28]. However, as the depth of the network increases, training results will get worse. ResNet can solve this problem by learning identity mapping. In order to balance the model performance and parameters, we finally adopt a 50-layer ResNet as basic model.

The proposed ResFi is inspired by FCN, CNN, and ResNet which are theoretically proved and experimentally validated as effective techniques in image classification. We will elaborate the structure of ResFi in this subsection.

#### 3.3.1. Stochastic Residual Block

According to [12], the identity block can be mathematically defined as

(7) $$\tilde{y}=ℱ\left(\tilde{x},w_{b}\right)+\tilde{x},$$

where $\tilde{x}$ and $\tilde{y}$ are the vectors of input and output layer, respectively. $w_{b}$ are the weights of convolutional kernels, and $ℱ\left(\tilde{x},w_{b}\right)$ represents the residual mapping to be learned. The operation $ℱ+\tilde{x}$ is performed by a shortcut connection and element-wise addition.

Once the dimensions of $\tilde{x}$ and ℱ are unequal, a convolutional layer $w_{s}$ is added to the shortcut connections

(8) $$\tilde{y}=ℱ\left(\tilde{x},w_{b}\right)+w_{s}\tilde{x},$$

Inspired by “Dropout” [29], we add the randomicity to the shortcut connections. The identity and convolutional block can be rewritten as

(9) $$\tilde{y}=ℱ\left(\tilde{x},w_{b}\right)+B⊙\tilde{x},$$

(10) $$\tilde{y}=ℱ\left(\tilde{x},w_{b}\right)+B⊙w_{s}\tilde{x},$$

where B is a matrix which has the same dimension with $\tilde{x}$ and $w_{s}\tilde{x}$. Each dimension of B obey Bernoulli Distribution. We replace each residual block by stochastic residual block. Since the residual connections are randomly preserved, the stochastic residual block has the same function as Dropout, such as improving the model generalization ability and preventing overfitting.

#### 3.3.2. Long-Range Stochastic Shortcut Connection

**Figure 6:**
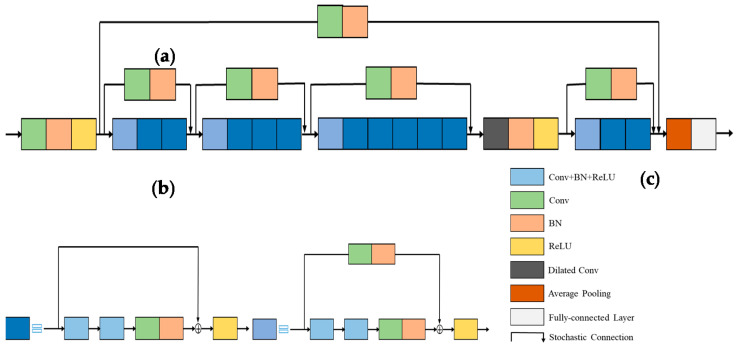
The network structure of ResFi. (**a**) ResFi; (**b**) stochastic identity block; (**c**) stochastic convolutional block.

Veit et al. [30] proposed a novel analysis that the residual networks can be interpreted as ensembles of many paths of differing length, instead of a single ultra-deep network. Inspired by the aforementioned identity and convolutional block, we propose the long-range stochastic shortcut connection to enhance the ensemble behavior, which can further mitigate the impact of network degradation and gradient vanishing. As shown in Figure 6a, the long-range stochastic shortcut connection can combine the low-level feature maps with deep layers. When the shallow layers have learned a desired residual mapping, the deep layers of ResFi can retain the feature mapping of shallow layers well. The LRSSC can also help to propagate the gradients from deep layers to shallow layers well. We build the LRSSC referred by (4.5). Since the dimensions of shallow and deep layers are unequal, we add a convolutional layer to the LRSSC. As shown in Figure 6a, there are 5 LRSSCs in ResFi. Specially, all the LRSSCs combine the shallow layer with the deep layer by a concatenation operation instead of element-wise addition. Thus, we can prevent losing information from previous layers and learn more feature maps by increasing the number of channels.

#### 3.3.3. Dilated Convolution

As shown in Figure 6a, different from the original ResNet architecture with two pooling layers, we only preserve the average pooling to avoid losing too much information of CSI image at the front of ResFi.

**Figure 7:**
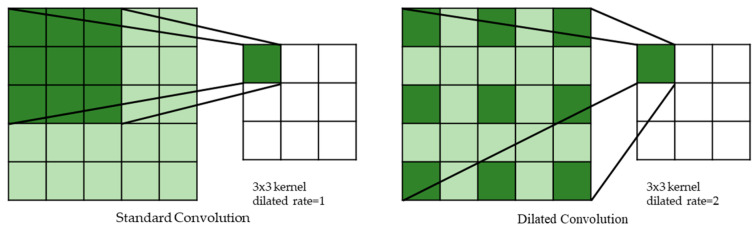
The comparison of standard and dilated convolution.

Since the pooling layers will lose information when the receptive field is enlarged. We adopt a dilated convolution [31] to increase the receptive field instead of the pooling layer. The dilated convolution by increasing the interval of weights in kernel obtains a larger receptive field without additional parameters. The dilation rate is set as two, and the comparison of standard convolution and dilated convolution is shown in Figure 7. Hence, the 3×3 kernel can obtain a 5×5 receptive field. Although dilated convolution is usually used in semantic segmentation, it is also effective in CSI image classification, and this will be testified in the experiments later. To reduce computation and memory, we put the dilated convolution in the rear of ResFi. In the actual implementation, one dilated convolution is enough to obtain sufficient effective receptive field.

### 3.4. Training Scheme

In order to train the network, the cross-entropy [32] with a regularization term is selected as the loss function to minimize the loss between the predicted label and ground truth label. The loss function $L_{R}\left(w\right)$ can be written as

$$L_{R}\left(w\right)=\frac{1}{2N}\sum_{i=1}^{N}\sum_{j=1}^{K}1\left\{z^{\left(i\right)}=j\right\}\log\frac{e^{w_{j}^{T}{\hat{x}}^{\left(i\right)}}}{\sum_{l=1}^{K}e^{w_{l}^{T}{\hat{x}}^{\left(i\right)}}}$$

(11) $$+\frac{1}{2}\sum_{i=1}^{N}\sum_{j=1}^{K}w_{\mathrm{ij}}^{2},$$

where N is the size of input training set. K is the total number of output neurons which is equal to the number of locations. 1{⋅} is the indicator function. $z^{\left(i\right)}$ is the index of the location of the ith CSI image and j is the index of output neurons. ${\hat{x}}^{\left(i\right)}$ is the output of second last layer and $w_{j}$ is the weight vector connecting the neurons in the second last layer to the output layer.

In the training stage, by minimizing $L_{R}\left(w\right)$ iteratively with momentum optimizer [33], we can optimize the network parameters w. In the testing stage, for a clean CSI image $x^{*}$, we feed it into the ResFi network and adopt the output of the fully-connected layer as the optimized deep image features. Then, we can obtain the estimated location by using Softmax classifier.

The pseudocode for weight training of our system is given in Algorithm 1. The inputs of Algorithm 1 are CSI images from all training locations, location labels, max iterations and learning rate. Firstly, a set of image pairs are generated by Bernoulli Sampling. For each iteration, we decrease the weights θ by descending the stochastic gradient. Then, we can get a clean image by removing the noise from the noisy image. After the weights training of Denoiser, we randomly select a mini-batch of N training samples and feed them into ResFi. Finally, the weights w are updated by descending the stochastic gradient.
**Algorithm 1** Weights Training of the Denoiser and ResFi**Input:** a set of noisy images ***n***, labels *l*, max iterations of Denoiser *maxid*, max iterations of ResFi *maxir*, learning rate *α* and *β***Output:** Trained weights ***w***^∗^ //Weight training of DenoiserGenerate Bernoulli sampled image pairs of a noisy image: ${\left\{{\hat{n}}_{m},{\bar{n}}_{m}\right\}}_{m=1}^{M}$Randomly initialize ***θ*****for** *iteration* = 1: *maxid*
**do** Update the Denoiser by descending the stochastic gradient:$$\theta^{*}=\theta -\alpha \frac{\partial L_{D}\left(\theta \right)}{\partial \theta }$$**end**Obtain the clean image: $x^{*}={ℱ}_{\theta }\left({\hat{n}}_{m}\right)$
//Weight training of ResFi Randomly initialize *w*
**for** *iteration* = 1: *maxid*
**do** Randomly select a mini-batch of *N* training samples:
$\left\{x^{*}{}_{i},l_{i}\right\},\;i=1,\ldots ,N$Update the ResFi by descending the stochastic gradient:$w^{*}=w-\beta \frac{\partial L_{R}\left(w\right)}{\partial w}$**End** Obtain the optimal weights: ***w***^∗^


## 4. Experiments

### 4.1. Experimental Setup

Our CSI collecting equipment is composed of two parts, the access point and mobile terminal. We use a TP-Link wireless router as the AP which is responsible for continuously transmitting packets. A Lenovo laptop equipped with Intel 5300 network interface card serves as mobile terminal to collect raw CSI values. A desktop PC with NIVIDA RTX 2070 SUPER Graphic card serves as the model training servers (based on the Tensorflow framework and CUDA Tool kit 7.5).

**Figure 8:**
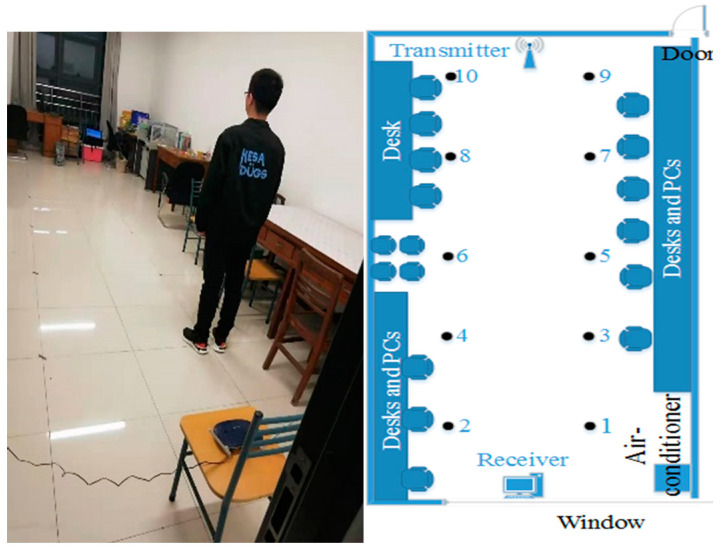
Training and test in the lab.

We conduct experiments to evaluate the performance of our system in a typical indoor scenario. As shown in Figure 8, this is a 4×10 m laboratory with some obstacles, such as desktop computers, chairs, and tables. The wireless router and PC are placed at the end of the area with the fixed height of 0.6m. We choose 10 locations (marked as black dots) to be tested. The raw CSI values are collected by CSI Tool [34] at each location. If the PC Pings the AP once, the AP will return a packet to the PC. In these experiments, we set the interval of Pings as 0.01 s and record with 5 min at every location. Thus, we obtain 30,000 packets at every location and then convert them into 1000 CSI images. Finally, the CSI images are increased to 63,000 by using data augment.

### 4.2. Analysis of the Experimental Parameters and Settings

In this subsection, we empirically evaluate the impact of different parameters of ResFi and experimental settings.

#### 4.2.1. Impact of the Convolutional Kernel Size

**Figure 9:**
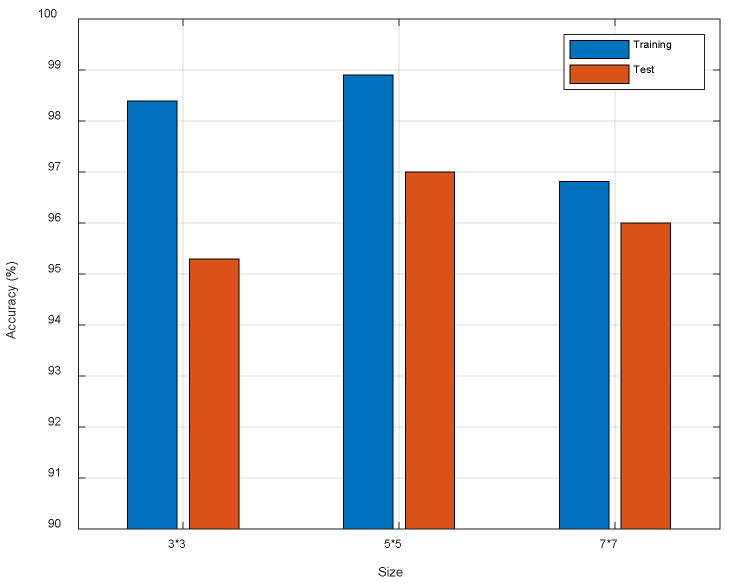
The comparison of different kernel size.

Since we need to match the dimensions of feature maps in the branches and backbone, the stride and size of convolutional kernels in branches need to be fixed first. Thus, we only analyze the impacts of kernels size in the backbone. Figure 9 shows the model performance with different kernels size. We find kernel 5×5 is the best choice, and this is because the kernel 5×5 is suitable for feature extraction of CSI images.

#### 4.2.2. Impact of the Number of Dilated Convolutions

**Figure 10:**
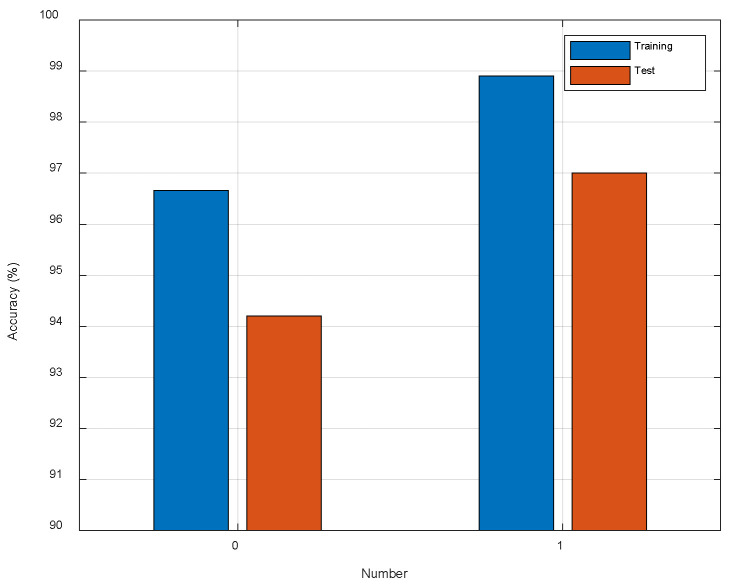
The comparison of different number of dilated convolutions.

As shown in Figure 10, we observe the test accuracy is improved about 2.80% with one dilated convolution. The result confirms that dilated convolution is effective for CSI image classification. The kernel size of dilated convolution is 3×3 with dilation rate of two. Compared to the pooling operation, the receptive field increases without losing spatial information, and this is undoubtedly beneficial for localization task. In addition, dilated convolution should be also suitable for other classification tasks.

#### 4.2.3. Impact of the Number of Convolutional Kernels

**Figure 11:**
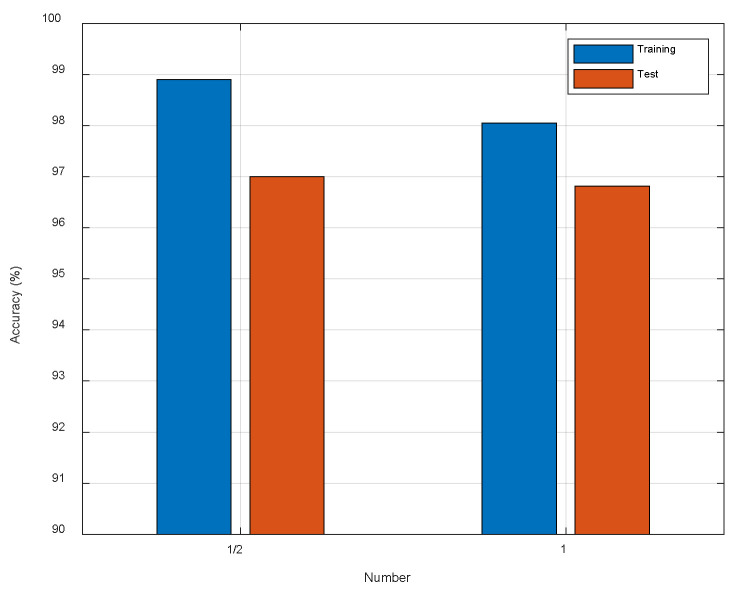
The comparison of different number of convolutional kernels.

As we know, more convolutional kernels require more computational cost. Therefore, we conduct some experiments to seek a suitable number of the convolutional kernels. Firstly, we set the number of convolutional kernels to be the same as the original ResNet. Then, we halve the number of convolutional kernels. As shown in Figure 11, as the number of convolution kernels has been halved, the localization performance has a subtle increase. This means that we do not need so many parameters, so we halve the number of convolutional kernels of ResNet-50 to reduce the computational cost.

#### 4.2.4. Impact of the Number of Iterations

**Figure 12:**
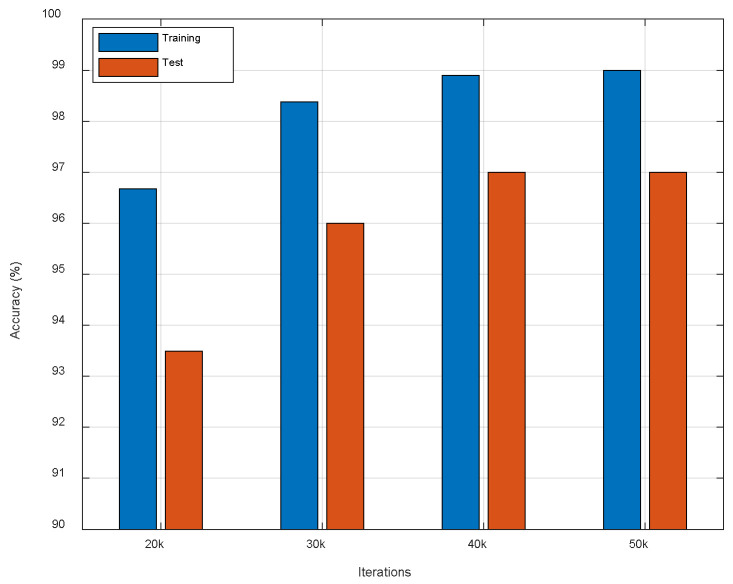
The comparison of different iterations.

Since proper iterations can prevent overfitting and reduce computational cost, we compared different iterations of the ResFi to seek a suitable one. Figure 12 shows that 400,000 iterations and 500,000 iterations get the best performance. This shows that the loss function has converged when the number of iterations is 400,000. Therefore, we choose 400,000 as the maximum iterations.

#### 4.2.5. Analysis of the Robustness

**Figure 13:**
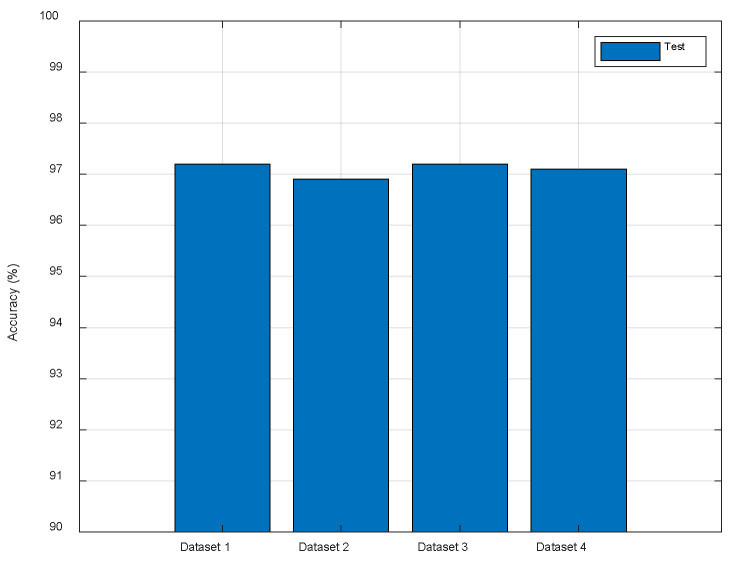
The comparison of different routers.

To test the robustness of our localization method to different routers, we construct Dataset 2 and 3 by using two additional TP-Link routers to measure the CSI data, respectively. In addition, we replaced the tester when we constructed Dataset 2. The original test dataset is named Dataset 1 and the combination of Dataset 1, 2, and 3 is named Dataset 4. In addition, the measurement environment of dataset 2 and 3 is a little different from that of dataset 1. As shown in Figure 13, ResFi performs stably on different Datasets which demonstrates that the proposed method is robust to different routers, a certain degree of environmental changes and the replacement of tester.

### 4.3. Ablation Experiments

**Figure 14:**
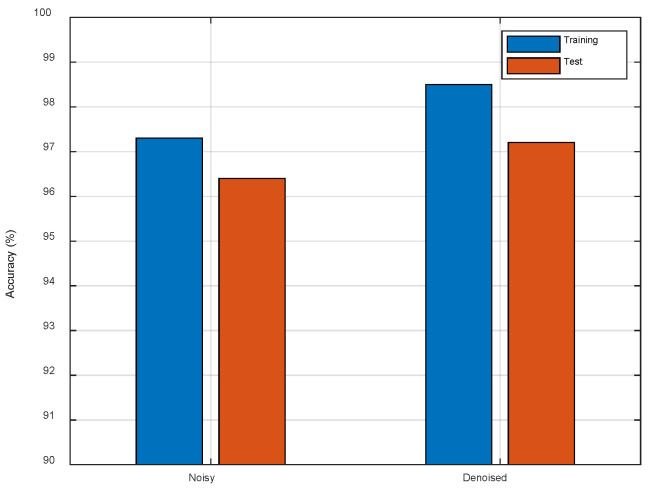
The comparison of different datasets.

To test the impact of the Denoiser, we use the originally noisy CSI images and denoised CSI images as the training data, respectively. As shown in Figure 14, we observe that the test accuracy is improved about 0.8% which demonstrates that the random noise has certain interference to the network. The denoised CSI images can improve the localization accuracy by preserving the main line features.

### 4.4. Comparison of the Existing Methods

We have compared ResFi with three existing NN based methods, including DANN, DeepFi and ConFi. The parameters of the algorithms are all tuned to give the best performance. Since the overfitting problem is serious in ConFi, we add a Dropout layer at the end of the network. For a fair comparison, all schemes use the same data set to estimate the position of the moving object.

We use mean error ℳ estimated on test dataset as the metric of localization performance. For M mistakenly estimated locations, $\left(a_{i}^{*},b_{i}^{*}\right)$ represents the estimated location of objection i, and $\left(a_{i},b_{i}\right)$ represents the real location. The mean error is defined as

(12) $$ℳ=\frac{1}{M}\sum_{i=1}^{M}\sqrt{{\left(a_{i}^{*}-a_{i}\right)}^{2}+{\left(b_{i}^{*}-b_{i}\right)}^{2}}.$$

**Figure 15:**
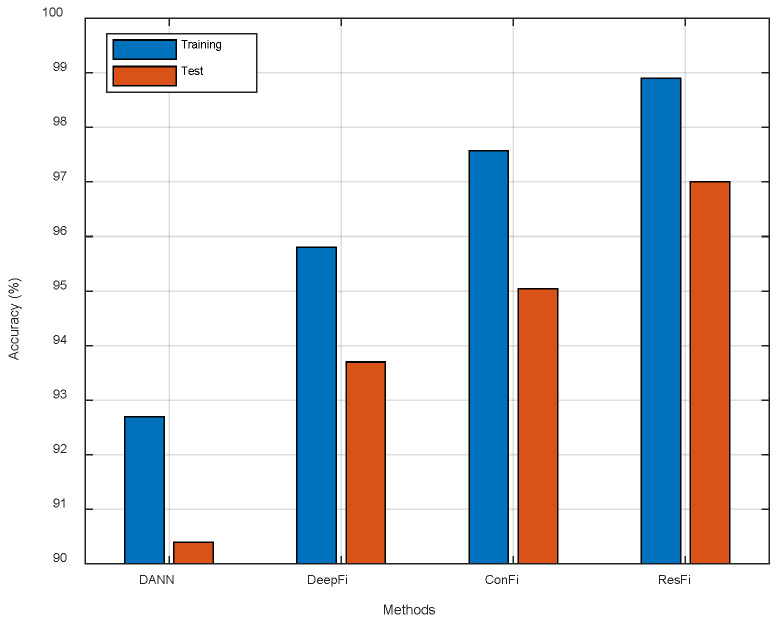
The comparison of different methods.

As shown in Table 1, we provide the mean error and the standard deviation of localization errors. Our system achieves the mean error of 1.7873 m and the standard deviation of 1.2806 m. It indicates that ResFi-based indoor localization is the most precise in these methods. ResFi also shows robust performance for different locations with the smallest standard deviation. As shown in Figure 15, compared to ConFi, ResFi improves the localization accuracy about 1.96%. In the actual experiments, ResFi outperforms the other three schemes in localization accuracy.

**Figure 16:**
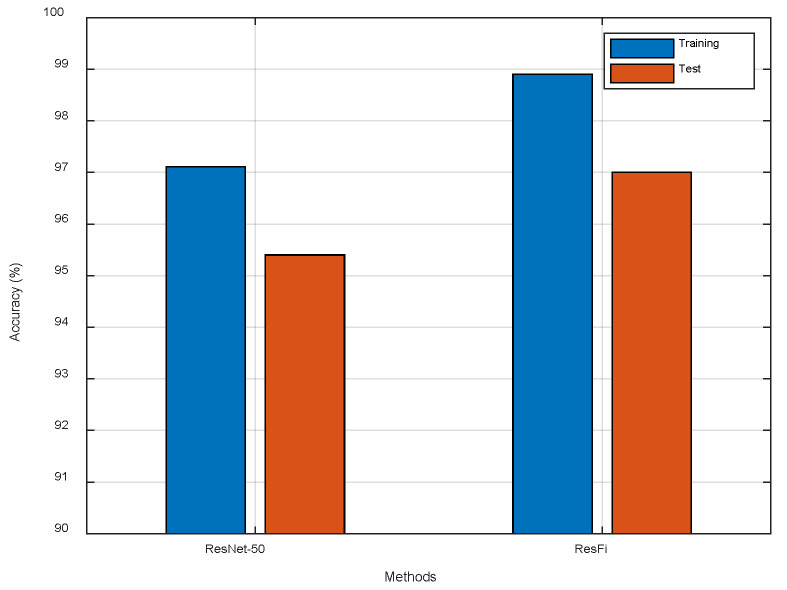
The comparison of ResNet-50 and ResFi.

We also apply ResNet-50 to indoor localization in another experiment. The results are illustrated in Figure 16. Compared to ResNet-50, ResFi improves the localization accuracy about 1.6%, which indicates that ResFi can extract more effective features from CSI images than ResNet-50.

## 5. Conclusions

In this paper, we proposed a denoising NN and a novel ResNet architecture to classify the CSI images. By full use of the low-level features in the deep layers of the denoising NN, we could improve the denoising performance and reduce the parameters. Moreover, the stochastic residual block was proposed to effectively prevent overfitting. Specially, the long-range stochastic shortcut connection was used to further boost information propagation between shallow and deep layers. Through empirical validation and analysis, ResFi was proved to achieve significant improvement in indoor localization. The experimental results also confirm that ResNet has better performance in indoor localization than CNN. However, the indoor localization of multiple objects is still a challenging task which is worthy of further study in the future.

## Figures and Tables

**Table 1:** The comparison of localization error.

Algorithm	Mean Error (m)	Std.dev. (m)	Parameters (M)
DANN	2.3910	1.6507	0.85
DeepFi	2.1082	1.4821	1.76
ConFi	1.9365	1.3554	8.11
ResFi	1.7873	1.2806	14.07

## Data Availability

The data are available at https://github.com/Jacriper/ResFi (accessed on 30 April 2021).
